# The pollination of *Habenaria rhodocheila* (Orchidaceae) in South China: When butterflies take sides

**DOI:** 10.1002/ece3.7242

**Published:** 2021-03-02

**Authors:** Xing‐Hui Chen, Shao‐Lin Tan, Yue‐Long Liang, Lang Huang, Han‐Wen Xiao, Huo‐Lin Luo, Dong‐Jin Xiong, Bo‐Yun Yang, Zong‐Xin Ren

**Affiliations:** ^1^ Jiangxi Key Laboratory of Plant Resources School of Life Sciences Nanchang University Nanchang China; ^2^ Jiulianshan National Natural Reserve Administration Bureau Ganzhou China; ^3^ CAS Key Laboratory for Plant Diversity and Biogeography of East Asia Kunming Institute of Botany Chinese Academy of Sciences Kunming China

**Keywords:** breeding system, butterflies, *Habenaria*, pollination efficiency, reproductive success

## Abstract

*Habenaria* is one of the largest terrestrial genera in the family Orchidaceae. Most field studies on *Habenaria* species with greenish–white and nocturnal scented flowers are pollinated by nocturnal hawkmoths and settling moths. However, *H. rhodocheila* presents reddish flowers lacking a detectable scent and fails to fit the moth pollination syndrome. We investigated the pollinators, breeding system, and functional traits of *H. rhodocheila* in South China and found that two diurnal swallowtail butterflies *Papilio helenus* and *Papilio nephelus* (Papilionidae) were the effective pollinators. When butterflies foraged for nectar in the spur, the pollinia became attached between the palpi. A triangular projected median rostellar lobe was found at the entrance (sinus) of the spur of *H. rhodocheila*. This lobe divided the spur opening into two entrances forcing butterflies to enter their proboscides through the left or right side. When the projection of median rostellar lobe was removed, the site of pollinium attachment changed to the eyes of the butterflies, leading to a higher rate of pollinium removal but lower rate of pollinium deposition. Our quartz glass cylinder choice experiment suggested that visual rather than olfactory cues provided the major stimuli for butterflies to locate these flowers. Hand pollination experiments suggested this species was self‐compatible but pollinator‐dependent. However, the proportion of seeds with large embryos produced in self‐pollinated fruits was significantly lower than in cross‐pollinated fruits, indicating a significant inbreeding depression. Unlike many other orchid species, fruit set was higher than rates of pollinium removal, indicating a high level of pollination efficiency in a species with friable pollinia. Shifts from moth to butterfly pollination in the genus *Habenaria* parallel other orchid lineages providing insights into the potential for pollinator‐mediated floral trait selection.

## Graphical abstract

Two diurnal swallowtail butterflies *Papilio helenus* and *Papilio nephelus* (Papilionidae) were the effective pollinators of *Habenaria rhodocheila*. Visual rather than olfactory cues provided the major stimuli for butterflies to locate these flowers. The proportion of seeds with large embryos produced in self‐pollinated fruits was significantly lower than in cross‐pollinated fruits, indicating a significant inbreeding depression.

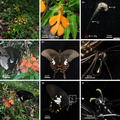

## 1 INTRODUCTION

Orchids consist of 736 genera and approximately 28,000 species forming the largest family of flowering plants (Chase et al., 2015; Christenhusz & Byng, 2016). Several hypotheses have been proposed to explain their great diversity (Givnish et al., 2015; Gravendeel et al., 2004). In particular, the hypothesis of the roles of pollinator‐driven selection to promote outcrossing in adaptive radiation has long been debated (Cozzolino & Widmer, 2005; Gravendeel et al., 2004; Micheneau et al., 2009; Tremblay et al., 2005; Van der Pijl & Dodson, 1966). Meanwhile, the hypothesis of a coevolutionary race between floral depth (including spur length) and the proboscis length of pollinators was first proposed by Darwin (1859). The importance of this key, floral trait was confirmed 135 years later (Alexandersson & Johnson, 2002; Balducci et al., 2019; Martins & Johnson, 2007; Nilsson, 1988; Wasserthal, 1997). However, the function of a spur matching a proboscis cannot be understood without the investigation of other signals and/or effective pollen transfer traits targeting pollinium attachment to the pollinator and receptive stigma lobes (Johnson et al., 2020; Pedron et al., 2012; Tao et al., 2018). Such studies remain uncommon (Johnson et al., 2020).

Orchids offering nectar rewards are pollinated primarily by insects in three Orders, Hymenoptera, Lepidoptera, and Diptera (Luo et al., 2020; Nilsson, 1992), although a few are pollinated by birds (Johnson, 1996; Johnson & Brown, 2004; Johnson & Van der Niet, 2019; Micheneau et al., 2006). The Order Lepidoptera shows one of the largest insect radiations with approximately 160,000 species, and butterflies represent 12% of species diversity in Lepidoptera (Kawahara et al., 2018, 2019; Mitter et al., 2017). Some orchid genera including *Bonatea* (Balducci, Martins, et al., 2019; Balducci et al., 2019) and *Habenaria* (Pedron et al., 2012) show functional traits compatible with Lepidoptera pollination. In these genera, most species have evolved a suite of floral traits that attract nocturnal moths, while a few species in the same lineage show suites adapted to diurnal butterflies. These lineages provide ideal model systems to understand the evolution of pollinator shifts.

*Habenaria* Willd. is one of the largest, terrestrial, orchid genera with about 928 species (Govaerts et al., 2019). It is distributed from temperate–tropical regions around the world, with centers of diversity in Brazil, southern and central Africa, and East Asia (Batista et al., 2013; Chase et al., 2015). The flowers of *Habenaria* species are characterized by two separate stigmatic lobes and each stigma lobe connects to a pollinium via a sterile caudicle. With important exceptions, their flowers are usually greenish‐whitish with a long nectar‐secreting spur (Zhang & Gao, 2018). The pollination ecology of the genus *Habenaria* has attracted attention since the 19th century (Guignard, 1886; Robertson, 1893), while studies on the efficiency of its pollinators and its breeding system began two decades ago (Singer, 2001; Singer & Cocucci, 1997). Since then, pollination biology of about 30 species of *Habenaria* has been studied. Earlier studies focused on identification of pollinators and the lengths of their proboscides versus spur length (Moreira et al., 1996; Thien, 1969). So far we know that *Habenaria* species are generally self‐compatible but pollinator‐dependent and pollinated by crepuscular‐nocturnal moths (Claessens et al., 2019; Peter et al., 2009a; Singer & Cocucci, 1997). Other studies confirmed that a few *Habenaria* species were pollinated by butterflies (Dangat & Gurav, 2014; Moreira et al., 1996; Pedron et al., 2012; Suetsugu & Tanaka, 2014), or rarely by Diptera (Singer, 2001; Thien, 1969), or a juvenile katydid (Suetsugu & Tanaka, 2014).

Competition for pollinium attachment sites on the same vector's body were strongly selected among species within this genus. The pollinia of *Habenaria* species are usually attached to smooth body parts especially eyes (Moreira et al., 1996; Singer & Cocucci, 1997; Thien, 1969) and the bases of the proboscides (Singer, 2001; Xiong et al., 2015). To a lesser extent, the pollinia may be deposited on legs (Peter et al., 2009a; Xiong et al., 2019), thoraxes (Johnson et al., 2020; Sakagami & Sugiura, 2019), or on bare areas of the head (Suetsugu & Tanaka, 2014), or between the palpi (Pedron et al., 2012). Worldwide field studies conducted in South America and Africa tend to predominate. Although 54 species are distributed in China (Chen & Cribb, 2009), *Habenaria* pollination has been reported in only six species in Yunnan province indicating that hawkmoths and/or other nocturnal moths were pollinators (Tao et al., 2018; Xiong et al., 2015, 2019; Zhang & Gao, 2017). No butterfly or other diurnal pollinators has been reported as *Habenaria* pollinators in China to date.

Based on a search of the literature, *Habenaria rhodocheila* is the first Asian species in this genus known to be pollinated by swallowtail butterflies (Papilionidae: *Papilio*) as reported from Thailand by Williams and Watthana (2011). A recent study in Guangxi Province, south China showed that the pollinator of *H. rhodocheila* was *Papilio helenus* (Zhang et al., 2021), a congeneric Thailand pollinator *Papilio memnon*. However, pollinator attraction mechanism and functional significance of floral traits on butterfly pollination in this species remains largely unexplored. *Habenaria rhodocheila* is distinctive from its Asian congenerics by its orange–yellow–red and seemingly scentless flowers which are indicative of dependence on diurnal pollinators. The preliminary study shows that the spur is long and contains nectar. Additionally, the structure of the median rostellar lobe at the entrance of the spur divides the entrance into two openings. While this architectural trait is unique to this lineage, its adaptive significance remains untested. Therefore, we addressed the following questions. (a) Do the pollinators in Thailand match those further north in China? (b) Does floral color and/or an undetected scent attract the pollinators? (c) Is this species self‐compatible and does a low rate of pollen deposition on receptive stigmas limit maternal fitness (proxy)? (d) Does the median rostellar lobe affect pollinator behavior influencing reproductive success?

## 2 MATERIALS AND METHODS

### 2.1 Study species and site

**FIGURE 1:**
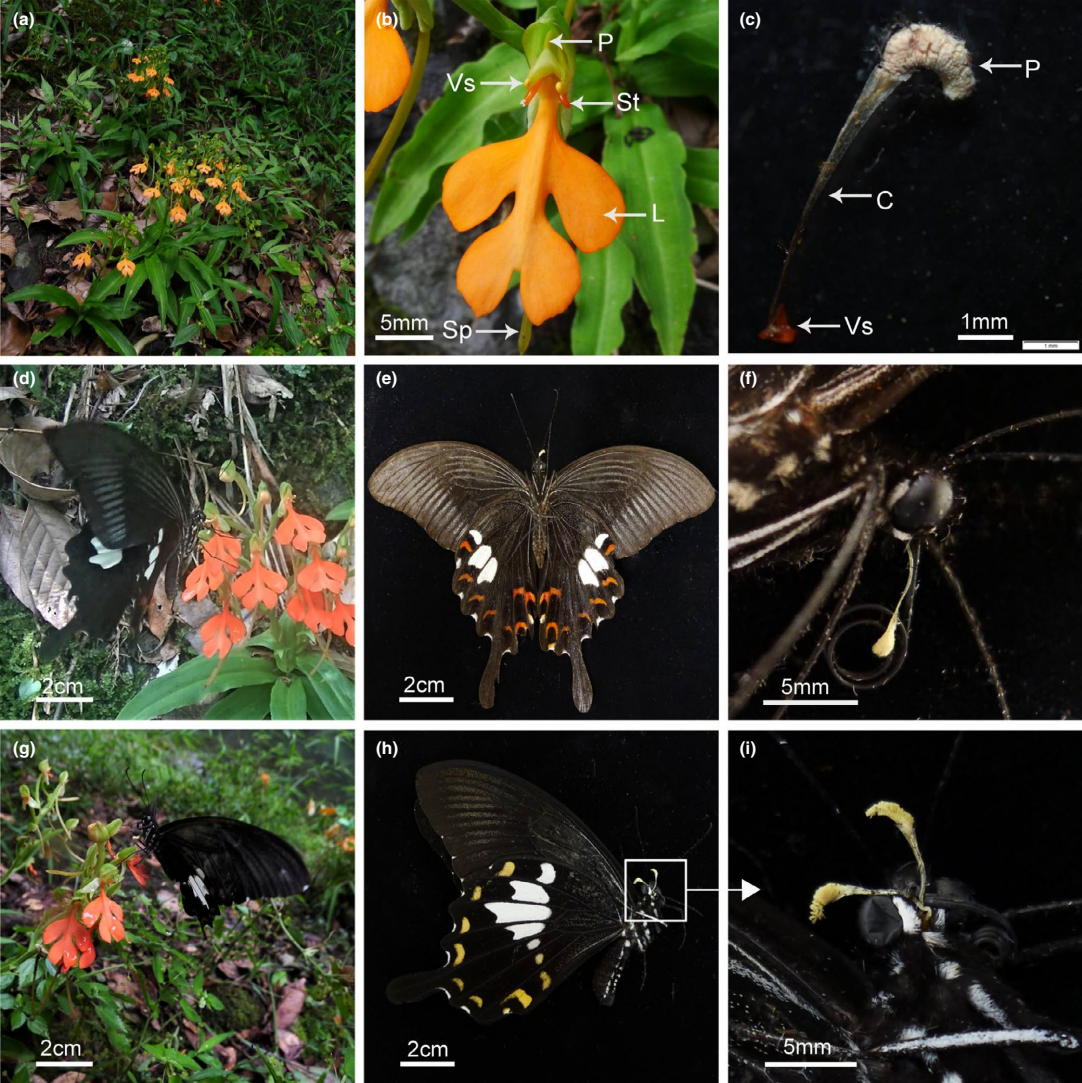
Habitat, flowers, and pollinators of *Habenaria rhodocheila* in Jiulianshan Mountain, South China. (a) Plants of *H. rhodocheila* growing on rocks in a subtropical forest margin. (b) A flower of *H. rhodocheila*: P, pollinium; Vs, Viscidium disk; St, Stigma; L, Labellum; Sp, Spur. (c) The pollinium of *H. rhodocheila*: Vs, Viscidium disk; C, caudicle; P, pollinium; (d) *Papilio nephelus* foraging *H. rhodocheila* flowers. (e,f) *Papilio Helenus* with pollinium of host flower attached between the palpi; (g) *Papilio helenus* visiting *H. rhodocheila* flowers. (h) *Papilio helenus* bearing pollinia of *H. rhodocheila* between its palpi; (i) Enlargement of the portion circled in (h)

*Habenaria rhodocheila* Hance (1866) is distributed from South China to Southeast Asia, and is characterized by three or more flowers/scape, an orange–yellow to red lip peal with a claw at its base, and two separate stigmatic lobes connected by a pollinium, respectively (Figure 1). It usually inhabits shaded places or soil‐covered rocks in forests or along valleys (Chen & Cribb, 2009).

The study site was located in the Jiulianshan National Nature Reserve, which is a part of the Nanling Mountains and on the border between the Provinces of Jiangxi and Guangdong. The Nanling Mountains form the transition zone between the middle and southern subtropical regions in China. The dominant vegetation type in this area is classified as subtropical evergreen broadleaved forests, and it is one of the biodiversity hotspots in east China (Tang et al., 2006). The coordinates of the study site are 114°30′E, 24°36′N (the detailed GPS data is not provided due to conservation purposes for endangered orchid species). The elevation ranged from 550 to 580 m. Voucher specimen (CXHHR001) was deposited in the Herbarium of the Nanchang University. The climate data for the period 1976–2015 were collected from the Xiagongtang weather station, located southwest of our study site. The mean annual temperature was 17.1°C, mean annual precipitation was 1,816.3 mm, and mean annual evaporation was 781.6 mm (Zhou & Liu, 2018).

### 2.2 Phenology, floral traits, and nectar properties

We studied floral phenology, floral traits, and nectar secretion of *H. rhodocheila* in situ from July to August in 2018. We randomly selected and tagged 30 inflorescences/individuals from the population to record phenology. This included documentation of the day when the first flower opened until the last flower withered. The opening day and withering day of each flower were recorded for each inflorescence.

We randomly selected one flower from each of the 30 inflorescences to measure seven floral traits using digital calipers (MNT‐150) with an accuracy of 0.01 mm. The following traits included: the length of spur, the distance between the spur opening (sinus) to the viscidium, the distance between the terminus of the spur to the viscidium, the distance between the two stigma lobes, the distance between the two viscidia, the length of caudicle (we measure one caudicle in each flower), the length and width of a pollinium (see Table 1 and Figure 1).

**TABLE 1:** Floral morphology of *Habenaria rhodocheila* on Jiulianshan Mountain, South China, with *N* = 30 flowers on 30 inflorescences used for trait measurements

Trait	Range (mm)	Mean (mm) ± *SD*
Length of spur	39.42–42.39	41.26 ± 1.02
Distance between opening of spur to the viscidium	5.08–6.36	5.58 ± 0.51
Distance between the terminus of the spur to the viscidium	45.31–48.23	47.21 ± 1.14
Distance between the two stigma lobes	2.01–3.89	2.94 ± 0.84
Distance between two viscidia	2.70–3.24	2.82 ± 0.23
Length of caudicle	4.64–5.91	5.28 ± 0.43
Length of pollinium	2.77–3.19	2.96 ± 0.18
Width of pollinium	0.73–0.92	0.82 ± 0.06

We randomly selected 20 inflorescences in bud and isolated the entire scape in an organza (mesh) bag. When all the flowers on the inflorescence opened, two flowers were randomly selected on each scape for nectar measurement at noon (12:00) and midnight (24:00). We measured the nectar at noon due to peak periods of pollinator activity observed in situ. The length of nectar inside the spur was measured with a digital caliper. Then the spur tip was cut off with a pair of scissors, and the volume of the nectar was extracted and estimated with a capillary tube with an inner diameter of 0.5 mm. Nectar concentration was estimated with a handheld sugar refractometer (LH‐T10, 0%–50%).

### 2.3 Pollinator observations

We observed the floral visitors of *H. rhodocheila* from July to August 2018. Diurnal floral visitors were observed from 8:30 a.m. to 16:30 p.m. over 15 days (120 hr). To view nocturnal visitors, we first selected 10 inflorescences at random. Each inflorescence was bagged at daytime (8:30 a.m.–16:30 p.m.) and the same bag was removed at dusk. The next morning, we checked for pollinium removal and deposition of pollen fragments on the receptive stigma.

Foraging behaviors of pollinators were recorded using a digital camera HDR‐AC3 (Ordro) that tracked visiting time, the number of flowers probed during each visit, and the amount of time each forager probed each flower on each inflorescence. Pollinators observed foraging on flowers were caught with butterfly nets. The number of pollinia attached to each specimen was counted. The proboscis of each specimen was carefully unrolled and measured with a digital caliper. Specimens were freed following data collection excluding two specimens for each identified species. They were euthanized, spread, pinned, labeled, and deposited in the Zoological Museum of Nanchang University. These specimens and photos were identified by Professor Xing‐Ping Liu at Jiangxi Agricultural University, China.

### 2.4 Testing visual and olfactory cues

**FIGURE 2:**
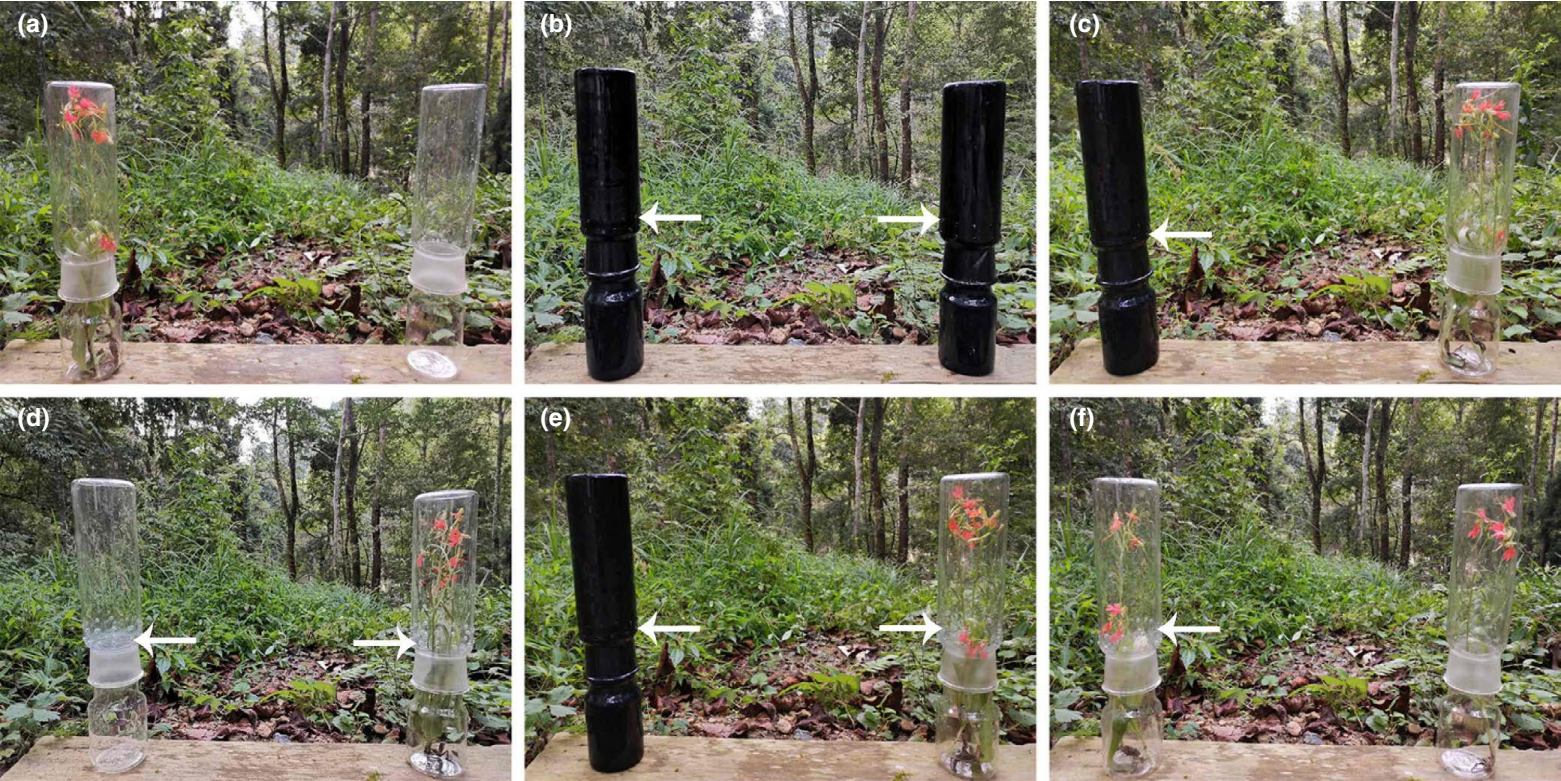
Quartz glass cylinders and *H. rhodocheila* inflorescences used to test the attractiveness of decoupled and combined visual and olfactory cues of *H. rhodocheila*. (a) Visual cues versus control; (b) Olfactory cue versus control; (c) Visual versus olfactory cue; (d) The combination of both visual and olfactory cues versus control; (e) Combination of visual and olfactory cues versus olfactory cues; (f) The combination of visual and olfactory cues versus visual cues. The arrows indicate the holes in the cylinder

We adopted the methods used by Burger et al. (2010) and Luo et al. (2020) under field conditions to test visual and olfactory signals for choice preference. In this experimental procedure, we used quartz glass cylinders and *H. rhodocheila* inflorescences to test the attractiveness of decoupled and combined visual and olfactory cues. A quartz glass cylinder consisted of a cap and body. Three types of cylinders were used in the experiment (Figure 2): (a) A black cylinder with holes for testing olfactory attraction only; (b) A transparent cylinder without holes for testing visual attraction only; (c) A transparent cylinder with holes for testing the combination of olfactory and visual attraction. In each experiment, we offered two cylinders to the butterflies.

Using cylinders and *H. rhodocheila* inflorescences, we conducted six bioassays (Figure 2): (a) visual cues (a transparent cylinder without holes containing an inflorescence) against a control (a transparent cylinder without an inflorescence); (b) olfactory cues (a black cylinder with holes containing an inflorescence) against a control (a black cylinder without an inflorescence); (c) visual cues against olfactory cues; (d) the combination of olfactory and visual cues (a transparent cylinder with holes containing an inflorescence) against a control; (e) the combination of visual and olfactory cues against olfactory cues; (f) the combination of visual and olfactory cues against visual cues. In each choice experiment, the cylinders were placed at a distance of 60 cm from each other. Each test was conducted for a total of 90 min. After 45 min, the position of the two cylinders was exchanged to account for position effects. The behavioral responses of butterflies in all bioassays were recorded each time a butterfly was observed flying towards the cylinder, and the minimum distance between the butterflies and the cylinders was less than 10 cm.

### 2.5 Median rostellar lobe removal experiment

**FIGURE 3:**
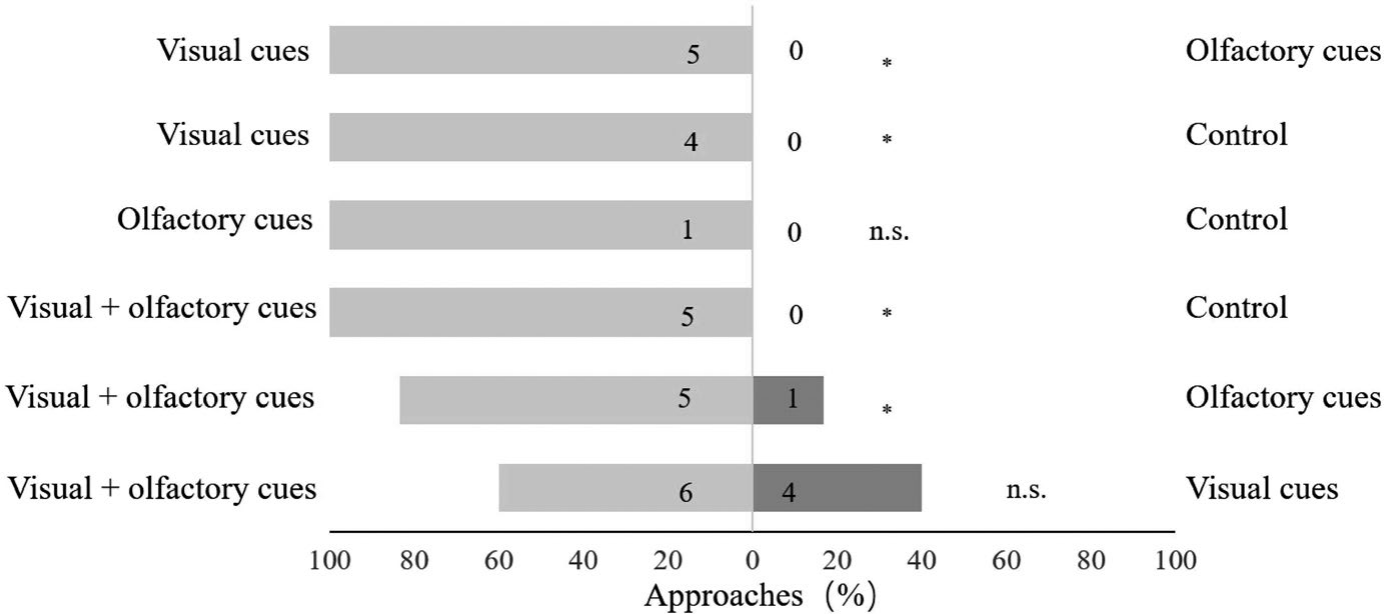
Comparative behavioral responses of butterflies to combinations of visual and olfactory cues of *Habenaria rhodocheila*. Numbers in bars are the absolute numbers of approaches (Binomial test: n.s., *p* > 0.05; *, *p* < 0.05)

To test whether the median rostellar lobe at the spur sinus could influence pollinator behavior and pollinium removal/pollinium deposition, we conducted the following experimental series (Figure 3). We randomly selected 30 flowers from fifteen plants and removing their median rostellar lobes with scalpel blade. We also selected 30 flowers at random from the same fifteen inflorescences as controls and did not remove their lobes. We observed the behavior of pollinators, caught them while they visited surgically altered flowers, recorded pollinium removal, and pollen deposition on flowers. These results were compared to observations of controls.

### 2.6 Pollination efficiency

To estimate pollination efficiency (PE), we examined pollinium removal and deposition in 30 flowers in our population during the later flowering stages. The PE was estimated following Scopece et al. (2010): PE = *F*_p_/*F*_r_, where *F*_p_ is the percentage of pollinated flowers and *F*_r_ is the percentage of flowers with one or two pollinia removed.

### 2.7 Breeding system

More than 30 inflorescences were bagged when the flowers were in bud and kept isolated until they were in full bloom. For each treatment, 30 flowers were selected at random. The treatments were as follows: (a) natural pollination ‐ flowers left intact allowing open, insect‐mediated pollination; (b) autonomous selfing—All flowers were covered with a mesh bag until all flowers on the scape wilted; (c) The anther was removed and the flowers were bagged; (d) Hand manipulated self‐pollination by applying the pollinia of a flower to its own two stigma lobes; (e) cross‐pollination by using pollinia from a flower at least 10 m away from the bagged inflorescence. The fruit set of each treatment was recorded in October, 2018.

### 2.8 Embryonic development

Ten dehiscent ovaries from each treatment (see above) were harvested to estimate embryonic development. All the seeds of each fruit were placed on a petri dish and examined under a light microscope, Olympus BX51 (Tokyo, Japan). Seeds were assigned to four categories following the method of Ren et al. (2014) i.e. large embryo, small embryo, aborted embryo, and no embryo. The inbreeding depression index (*δ*) was calculated following Suetsugu et al. (2015): *δ* = 1 − (proportion of well‐developed seeds after self‐pollination/proportion of well‐developed seeds after cross‐pollination). Seeds with large embryos were defined as well‐developed seeds.

### 2.9 Data analysis

We used the Mann–Whitney U test to estimate the difference in nectar volumes between day and night, and the differences between pollinium removal and pollinium deposition. A student *t*‐test was used to estimate the difference in nectar concentration between day and night, and the difference in pollinia numbers carried by two butterfly species. General linear models were performed to compare the difference in embryonic development among different pollination treatments. The exact binomial test was performed to test the differences in butterfly approaches to glass cylinders with different treatments. All analyses were implemented in R3.4.4 (R core team, 2018) and all ± values are presented as arithmetic mean ± standard deviation.

## 3 RESULTS

### 3.1 Phenology, floral traits, and nectar

Flowering time of the *H. rhodocheila* population lasted about 45 days, starting from the middle of July to the end of August. The flowering period of a single inflorescence was 16–18 days (*N* = 30), and the life span of a single flower was 12–13 days (*N* = 30). The inflorescence length ranged from 10 to 27.5 cm (22.13 ± 3.69 cm, mean ± *SD*, similar hereafter). The spur length ranged from 39.42 to 42.39 mm (41.26 ± 1.02 mm, Table 1). During daylight hours, the nectar length in the spur ranged from 9.76 to 17.20 mm with an average of 12.99 ± 2.24 mm. Extracted nectar volume ranged from 3.64 to 5.93 μl (4.38 ± 0.88 μl). The concentration of dissolved nectar solutes ranged from 17.5% to 23.0% (20.89 ± 2.04%). At night, nectar length in the spur ranged from 7.20 to 11.00 mm with an average of 9.27 ± 1.43 mm. Extracted nectar volume ranged from 3.23 to 5.06 μl (3.96 ± 0.74 μl). Concentration of dissolved solutes ranged from 17.0% to 24.5% (20.75 ± 2.27%). The nectar volume at noon did not differ from midnight recordings (Mann–Whitney *U* Test, *p* = 0.505, *N* = 20). Nectar concentrations taken at noon did not differ significantly from midnight (*t*‐test, *t* = 0.155, *p* = 0.881, *N* = 20).

### 3.2 Pollinators and their behavior

All 10 inflorescences bagged daytime hours, with bags removed at night failed to show pollinium removal and pollen deposition on stigmas, indicating the absence of nocturnal visitations. During daylight hours, we observed two species of swallowtail butterflies, *P. helenus* and *Papilio nephelus* (Papilionidae), visiting *H. rhodocheila* (Figure 1) foraging most often between 10:00 to 15:00. During the pollination process, a butterfly landed on the lip, and inserted its proboscis to either the right or left side of the sinus as entry was blocked by the median rostellar lobe (Video S1). As the length of the proboscides of the two *Papilio* species was shorter than the spur length these butterflies had to push their proboscides deep into the sinus to obtain more nectar. Therefore, both species pushed their heads against the column until the viscidia became attached to the region between their palpi (Figure 1). The butterflies probed 1–3 flowers per visit to each inflorescence. *Papilio helenus* spent 3–29 (12 ± 7.6) seconds at each flower and 4–32 (13.5 ± 8.3) seconds at each inflorescence. *Papilio nephelus* spent 4–20 (10.4 ± 5.6) seconds at each flower and 4–22 (13.8 ± 6.7) seconds at each inflorescence. All butterflies (ten for each species) caught visiting the flowers carried pollinia. Each individual of *P. helenus* carried 1–6 pollinia (3 ± 1.61, *N* = 10). Each individual of *P. nephelus* carried 2–4 pollinia (2.8 ± 0.75, *N* = 10). The difference between the numbers of pollinia carried by the two butterflies was not significant (*t* = 0.327, *df* = 9, *p* = 0.751). Proboscis lengths of *P. helenus* and *P. nephelus* were 37.21 ± 0.09 (*N* = 10) mm and 31.91 ± 0.22 (*N* = 10) mm, respectively.

### 3.3 Visual and olfactory floral cues

**FIGURE 4:**
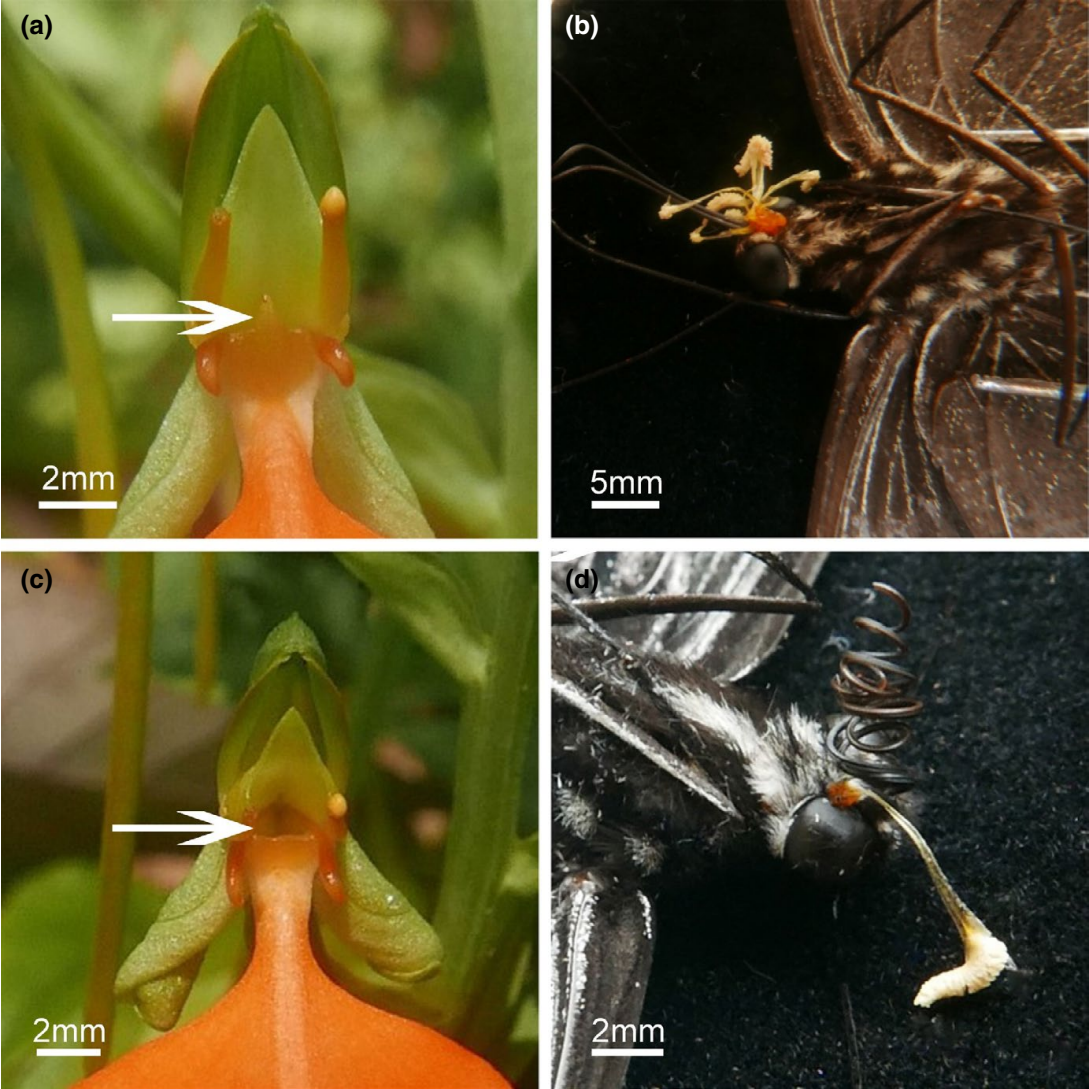
The median rostellar lobe removal experiments. The white arrow in figure A indicates the structure of the median rostellar lobe while the arrow in figure c indicates a flower in which the triangular projection was removed. In the natural control treatment (a), the pollinia were attached between the palpi of the pollinators (b). When the median rostellar lobe was removed (c), the pollinium was attached to the butterfly's eyes (d). When the median rostellar lobe was removed, the pollinium removal rate (86.66%) was higher than the natural control (75.30%) but pollinium deposition rate (70.00%) was lower than the natural control (83.33%)

Our experimental series showed that butterflies approached cylinders containing inflorescences ignoring the choice of the empty cylinder (Figure 4). When given an empty cylinder and a blackened cylinder containing an inflorescence with holes emitting the olfactory cue butterflies approached the scent secreting cylinder once, but never approached the empty cylinder. When given a cylinder with visual cues only and a second blackened cylinder with scent holes, butterflies preferred the visual cue cylinder versus the scent cylinder (*p* = 0.04). The presentation of visual and olfactory cues versus a visual cue did not show a significant difference (*p* = 0.13). These results indicated that visual presentation by inflorescences of *H. rhodocheila* was more likely to butterflies compared to the exclusive presentation of scent cues.

### 3.4 The effects of projected median rostellar lobe on pollination success

Pollinia were attached in between the palpi of butterflies visiting controls (see above, Figure 3a,b). When the median rostellar lobe was removed (Figure 3c) the pollinia were attached to the eyes of all three pollinators we caught and observed (Figure 3d). When the median rostellar lobe was removed, the pollinium removal rate (86.66%) was higher than in the controls (75.30%) However, with the lobe removed the pollinium deposition rate (70.00%) declined slightly compared with the control flowers (83.33%).

### 3.5 Pollination efficiency

Butterfly visitation rate of flowers in our study site was high. We found that 75.30% of flowers had at least one pollinium removed, and 83.33% of flowers received whole pollinium or fragments deposited on their stigmatic lobes. The value of pollination efficiency (PE) was 1.11.

### 3.6 Breeding system, fruit, and seed set

**FIGURE 5:**
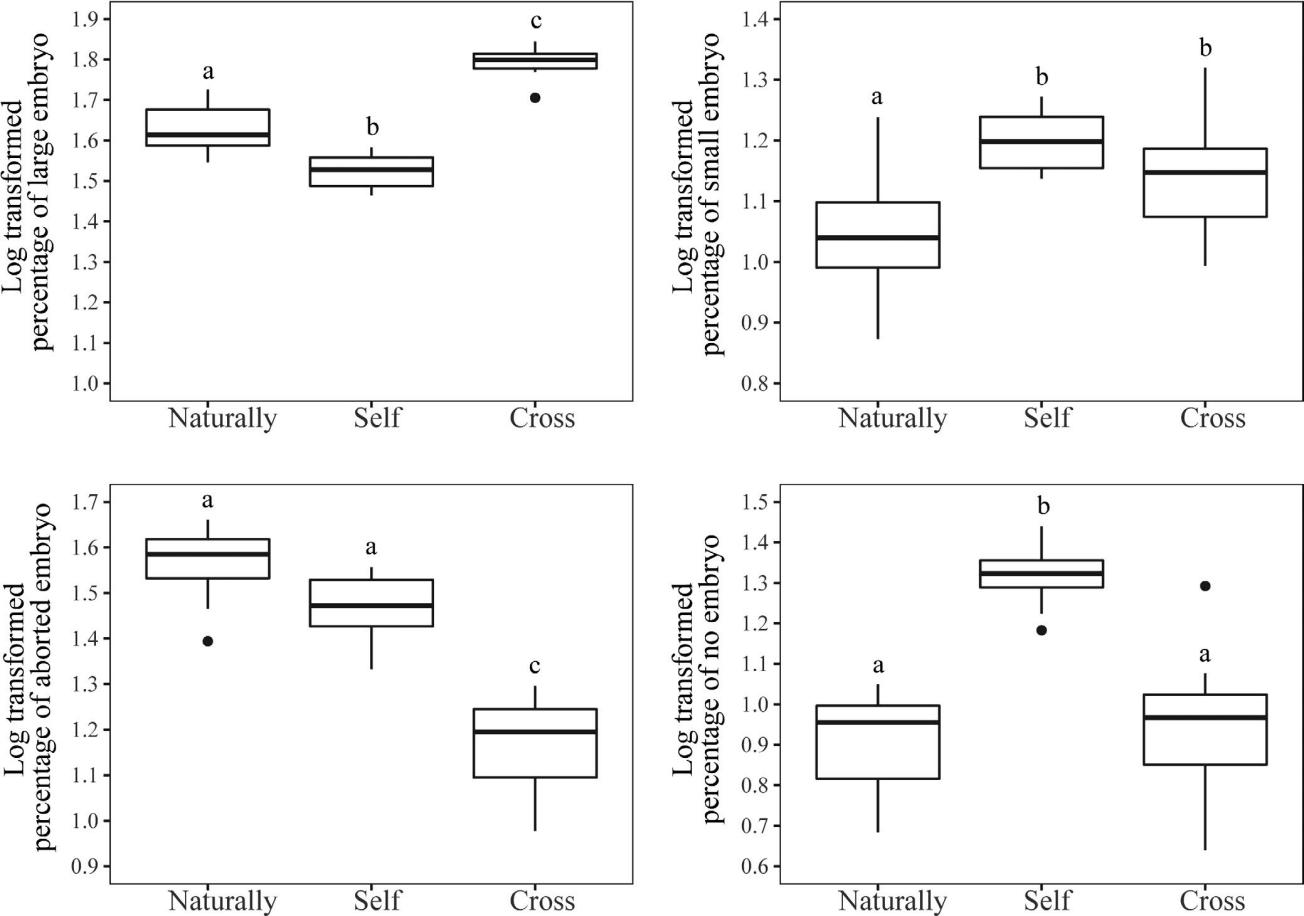
Comparative embryonic development in seeds produced by natural pollination, hand‐self, and hand‐cross‐pollination. Embryos were assigned to four categories: large (a), small (b), aborted (c), and no embryo (with empty testa) (d). All lower case letters indicate significant differences (*p* < 0.05)

Both bagged intact flowers and emasculated (anthers removed) flowers failed to produce fruits (Table 2). The fruit set of naturally pollinated, butterfly visited, flowers was 83.33%. Fruit set in self‐ and cross‐pollination series were 100%. However, the proportion of seeds with large embryos in fruits following cross‐pollination (62.26 ± 5.24%) was significantly higher than in the self‐pollination series (33.32 ± 3.60; Table 2; Figure 5). The proportion of seeds with large embryos in naturally pollinated fruits (42.67 ± 5.96%) was significantly higher than in self‐pollinated fruits but significantly lower than in hand‐mediated, cross‐pollinations. The proportion of seeds with aborted embryos in cross‐pollinated fruits was significantly lower than that of both self‐pollinated and naturally pollinated fruits. The inbreeding index (*δ*) was 0.219.

**TABLE 2:** Fruit set and embryonic development according to treatments within our study site

Treatments	Fruit set (%)	Proportion of seeds containing large embryos (%)	
		Range	Mean ± *SD*
Bagging	0	0	0
Emasculating	0	0	0
Self‐pollination	100	27.14–38.29	33.32 ± 3.60
Cross‐pollination	100	50.74–69.91	62.26 ± 5.24
Natural control	83.33	35.17–53.17	42.67 ± 5.96

Note

For each treatment, *N* = 30 flowers were used for the experiment with 10 fruits dissected for embryonic development.

## 4 DISCUSSION

Selection by pollinators is regarded as one of the main driving forces in floral evolution and speciation in orchids (Sletvold et al., 2010; Tremblay et al., 2005; Trunschke et al., 2017). This process is usually viewed as an asymmetric interaction in which floral traits change due to pollinator morphology and fidelity, while orchid flowers have a far lesser impact on pollinator evolution (Nilsson, 1992). *Habenaria* species show a classic suite of floral adaptations to nocturnal moth pollination long recognized historically by biologists (Faegri & van der Pijl, 1979). This includes white‐green flowers, longspurs with sugar‐rich nectar, crepuscular‐nocturnal emission of strong fragrances, and column modifications that contact moth anatomy uninterrupted by loose scales. In this study, however, *H. rhodocheila* showed a suite of traits most often associated with pollination by diurnal Lepidoptera. This is the first study to apply cylinder experiments to a *Habenaria* species confirming that visual cues are more important to the primary pollinators than scent. We also tested the functional morphology of the mid‐rostellar lobe so prominent in this genus and its allies. In *H. rhodocheila* this structure appears to influence rates of reproductive success as its presence canalizes movements of the primary pollinators.

### 4.1 Phenology, floral traits, and pollinator behavior

The flowering time period of our *H. rhodocheila* population lasted half a month longer than that of the Guangxi populations, which are about 420 km away from our study site (Zhang et al., 2021). The life span of a single flower in our population was also much longer than that of the Guangxi populations. The great differences in phenology between populations of the two study sites might be attributed to their apparent differences in climate variables and their genetic backgrounds. Compared with the Guangxi populations, our study site has lower temperature, more rainfall, and much less evaporation. The unique pollinator *P. helenus* in the Guangxi populations was also the pollinator in our study site. However, another congeneric pollinator *P. nephelus* in our study site was not determined as an effective pollinator in the Guangxi populations. The pollinator shifts among the Thailand, Guangxi, and Jiangxi populations of *H. rhodocheila* are likely determined by the local assemblages of potential pollinators, the match between the spur length of the orchids and proboscis length of pollinators, and pollinator behaviors. Interestingly, the mean proboscis length of our *P. helenus* population was approximately 10 mm longer than that of the Guangxi population, and the spur length of our *H. rhodocheila* population was also approximately 10 mm longer than that of the Guangxi population.

Our sampling showed that nectar concentrations in the spur of *H. rhodocheila* were similar to other *Habenaria* species. With some important exceptions, previous studies suggest that nectar concentration does not vary too much among *Habenaria* species (Pedron et al., 2012; Tao et al., 2018; Zhang & Gao, 2017). The exception to the rule is *H. pleiophylla* with a sugar concentration of 40%, twice higher than most other species (Singer et al., 2007). The few other *Habenaria* species pollinated by butterflies (e.g., *H. pleiophylla*, *H. montevidensis*, and *H. radiata*) emit discernible fragrances (Moreira et al., 1996; Pedron et al., 2012; Suetsugu & Tanaka, 2014), including foetid odours (e.g., *H. foliosa* var. *foetida*) during the daytime (Dangat & Gurav, 2014). However, floral traits expressed by *H. rhodocheila* are indicative of traditional pyschophily (Faegri & van der Pijl, 1979). This includes the bright red–orange and spreading lip providing landing platform, and the lack of noticeable fragrance. In addition, the spur length of *H. rhodocheila* was longer than the proboscides of both butterfly species forcing the pollinators to ram their heads against the column to reach rewards deep in the spur. This is indicative of pollination by long‐tongued Lepidoptera in general (Micheneau et al., 2014 ) and common in other *Habenaria* species (Pedron et al., 2012; Singer & Cocucci, 1997; Tao et al., 2018; Xiong et al., 2019; Zhang & Gao, 2017). Pollinium attachment between the palpi of butterflies also occurred in the genus *Bonatea s.l*. (Balducci, Van der Niet, et al., 2019), now submerged within *Habenaria s.s*. bas (Batista et al., 2013).

### 4.2 Visual and olfactory cues for attraction

Previous studies showed that *Habenaria* species produced fragrances to attract pollinators (Pedron et al., 2012; Peter et al., 2009a; Tao et al., 2018). However, while butterflies respond to a combination of visual and olfactory cues to locate flowers (Schäpers et al., 2015), our experiments indicated that the floral color of *H. rhodocheila* far overshadowed the role of scent as the main attractant. Similar results have been reported in a previous study on two *Calanthe* species, in which butterflies also used visual cues to locate flowers under dense forests (Luo et al., 2020). Swallow butterflies represent a well‐known family of butterflies, in which *Papilio* is the most species‐rich taxa (Allio et al., 2020). Recent studies showed that *Papilio* butterflies have a sensitive wavelength discrimination system which enhanced their ability to detect flowers in complex environments (Kinoshita & Stewart, 2020; Yoshida et al., 2015) such as the understory of the subtropical evergreen forests in south China.

### 4.3 The role of the median rostellar lobe

Obviously, flowers pollinated by night moths or diurnal butterflies must share some characters. This includes the long floral tube for promoting pollen transfer by Lepidoptera with proboscides longer than most bees, flies, or beetles. The impact of floral spur length on pollen dispersal/deposition has attracted the attention of evolutionary biologists since Darwin (1862). More recent studies manipulate spur lengths of orchids using artificial spurs (Johnson & Steiner, 1997; Trunschke et al., 2020). However, the role of the narrow sinus offering access to proboscides has been neglected. As an example, in the genus *Calanthe*, butterfly‐pollinated species also have narrow entrances forcing butterfly proboscides to make contact with viscidia (Luo et al., 2020). This is definitely not the case in column‐spur architecture in *Habenaria* species and allied genera like *Platanthera* (Robertson & Wyatt, 1990). Each column subdivides its anther releasing two separate pollinium receiving pollen at two subdivided stigmatic lobes. In fact, different *Habenaria* species, present their pollinia to different sites on the bodies of Lepidoptera (Johnson et al., 2020; Pedron et al., 2012; Tao et al., 2018). Therefore, what the flowers of different *Habenaria* species have in common is that their segregating, bi‐lobed placement of stigma and pollinium actually increase the size of the spur sinus. Without the barrier of the mid‐rostellar lobe the slender proboscis of a butterfly could forage for nectar without its head ever contacting either viscidia or stigma or the pollinia could become affixed to the wrong part of the pollinator's body to permit pollinia contact with a receptive stigma.

The median rostellar lobe at the sinus of the spur was found in other *Habenaria* species (Kurzweil, 2009; Pedron et al., 2012; Ponsie et al., 2007) and in allied genera including *Bonatea* (Johnson & Liltved, 1997) and *Satyrium* (Johnson, 1997). These authors also suggested that the lobe forces pollinators to choose either side of the sinus. Consequently, only one of the two pollinia are removed at a time (Johnson & Liltved, 1997) unless the insect recoils its proboscis and pushes it into the opposite side of the sinus during the same visit. Similar with other cases such as *Bonatea* (Johnson & Liltved, 1997) and *Satyrium* (Johnson, 1997), *H. rhodocheila* also represents a reversal away from the “all eggs in one basket” gamete packaging strategy of most orchids. The results of our study confirmed the lobe's impact on pollinator behavior and on the deposition location of viscidia. Without the lobe, pollinium placement moves from the area between palpi and onto the eyes as in other species (see Pedron et al., 2012). Why is this important? If the site for viscidium deposition on the butterfly changes the rate of pollinium dispersal in *H. rhodocheila* appears unaffected but the rate of pollen deposition onto receptive stigmatic appears to declines. Forcing butterflies to forage from “side‐to‐side” increases pollen deposition on stigmas in *H. rhodocheila* and some of its congenerics (Pedron et al., 2012) often far higher than fertilization rates in other orchid lineages (Pedron et al., 2012; Tao et al., 2018). It appears that column architecture in *H. rhodocheila* and *Habenaria* species, in general, is better canalized and increases maternal fitness. Further research on variation in median‐rostellar lobe morphology in *Habenaria* species is needed to better test the relevance of morphological variation and its roles in competition for pollinium attachment sites and interspecific isolation where taxa are sympatric and co‐blooming.

### 4.4 Breeding system, inbreeding depression, and reproductive success

Fruit set in our population of *H. rhodocheila* was relatively high and similar to other field studies of species in this genus including *H. hieronymi* (Singer & Cocucci, 1997), *H. fordii* (Zhang & Gao, 2017), and *H. aitchisonii* (Xiong et al., 2019). However, very low fruit set is also known in *Habenaria* (Ikeuchi et al., 2015; Singer & Cocucci, 1997; Thien & Utech, 1970). We attribute high fruit set in *H. rhodocheila* to a combination of the rewarding nectar, bright easily detected flowers and, of course, to the dependable frequency and fidelity of the two species of pollinators. There was no evidence of apomixis or autonomous self‐pollination in our population providing additional evidence that *H. rhodocheila* is butterfly dependent. Similar results were found in the Guangxi *H. rhodocheila* populations (Zhang et al., 2021), as well as other *Habenaria* species based on similar breeding system experiments (Pedron et al., 2012; Singer, 2001; Tao et al., 2018; Zhang et al., 2017), with the exception of *H. malintana* (Zhang & Gao, 2018). Furthermore, most *Habenaria* species also showed a similar high level of fruit set in populations with residential pollinators (Pedron et al., 2012; Singer et al., 2007; Xiong et al., 2019). Only a few species showed less than a 60% rate of natural fruit set (Thien & Utech, 1970; Xiong et al., 2015). The usual interpretation attributes high fruit set in *Habenaria* species to consistent nectar rewards. In fact, higher visitation rates of pollinators to nectariferous orchids resulting in higher fruit set rates in nectariferous orchids than in nectarless species (Edens‐Meier et al., 2014; Gill, 1989; Neiland & Wilcock, 1998). As almost all of the hand self‐pollinated and cross‐pollinated *H. rhodocheila* flowers produced fruits, our results were consistent with previous studies on breeding systems in the genus (Pedron et al., 2012; Singer, 2001; Tao et al., 2018).

However, the results of embryonic development following different pollination treatments in *H. rhodocheila* also suggest that, although nectar rewards could increase pollination efficiency and fruit set it may also lead to higher levels of geitonogamy and mating between siblings or offspring with parents. In this species, this appears to produce a lower proportion of viable seeds as in *H. limprichtii* (Tao et al., 2018) and other orchid species (Peter & Johnson, 2009b). Our results of cataloging embryo development in hand‐cross, hand‐self, and insect‐pollinated flowers indicated that butterfly‐mediated self‐pollination was common in this *H. rhodocheila* population. Concentrated nectar rewards encouraged butterflies to visit more than one flowers on the same inflorescence. This permitted vector‐mediated geitonogamy as caudicles dried and re‐position the pollinia towards stigmatic surfaces.

A nectar addition experiment on a non‐rewarding orchid found that such rewards could significantly increase rates of pollinium removal but also geitonogamous self‐pollination (Johnson et al., 2004). However, the inbreeding index (*δ*) of *H. rhodocheila* was much lower than that of *H. limprichtii*, suggesting that inbreeding depression in *H. rhodocheila* was less severe than in *H. limprichtii*. Self‐compatibility is dominant in *Habenaria* (Pedron et al., 2012; Singer & Cocucci, 1997; Tao et al., 2018) and in the Orchidaceae in general (Micheneau et al., 2009; Tremblay et al., 2005). This may be a trade‐off, though as cross‐pollination in most orchids results in a dramatic increase in well‐developed seeds (Jersakova et al., 2006).

## CONFLICT OF INTEREST

Authors have no competing interests to declare.

## AUTHOR CONTRIBUTIONS

**Xing‐Hui Chen:** Data curation (lead); investigation (lead); methodology (lead); visualization (lead); writing – original draft (supporting). **Shao‐Lin Tan:** Funding acquisition (equal); investigation (supporting); writing – original draft (lead); writing – review and editing (lead). **Yue‐Long Liang:** Investigation (supporting); resources (supporting). **Lang Huang:** Investigation (supporting). **Han‐Wen Xiao:** Investigation (supporting); methodology (supporting). **Huo‐Lin Luo:** Investigation (supporting). **Dong‐Jin Xiong:** Investigation (supporting). **Bo‐Yun Yang:** Conceptualization (lead); funding acquisition (lead); investigation (supporting); project administration (lead); resources (lead). **Zong‐Xin Ren:** Conceptualization (lead); funding acquisition (equal); supervision (lead); writing – review and editing (lead).

## Supporting information

Video S1

## Data Availability

The data have been deposited in the Dryad and are accessible under https://doi.org/10.5061/dryad.pc866t1n8.
